# Modeling Subjective Affect Annotations with Multi-Task Learning

**DOI:** 10.3390/s22145245

**Published:** 2022-07-13

**Authors:** Hassan Hayat, Carles Ventura, Agata Lapedriza

**Affiliations:** Department of IT, Multimedia and Telecommunications (IMT), Universitat Oberta de Catalunya, 08018 Barcelona, Spain; alapedriza@uoc.edu

**Keywords:** supervised learning, aggregated annotations, emotion modeling, multitask learning, subjective labels

## Abstract

In supervised learning, the generalization capabilities of trained models are based on the available annotations. Usually, multiple annotators are asked to annotate the dataset samples and, then, the common practice is to aggregate the different annotations by computing average scores or majority voting, and train and test models on these aggregated annotations. However, this practice is not suitable for all types of problems, especially when the subjective information of each annotator matters for the task modeling. For example, emotions experienced while watching a video or evoked by other sources of content, such as news headlines, are subjective: different individuals might perceive or experience different emotions. The aggregated annotations in emotion modeling may lose the subjective information and actually represent an annotation bias. In this paper, we highlight the weaknesses of models that are trained on aggregated annotations for modeling tasks related to affect. More concretely, we compare two generic Deep Learning architectures: a Single-Task (ST) architecture and a Multi-Task (MT) architecture. While the ST architecture models single emotional perception each time, the MT architecture jointly models every single annotation and the aggregated annotations at once. Our results show that the MT approach can more accurately model every single annotation and the aggregated annotations when compared to methods that are directly trained on the aggregated annotations. Furthermore, the MT approach achieves state-of-the-art results on the COGNIMUSE, IEMOCAP, and SemEval_2007 benchmarks.

## 1. Introduction

**Figure 1:**
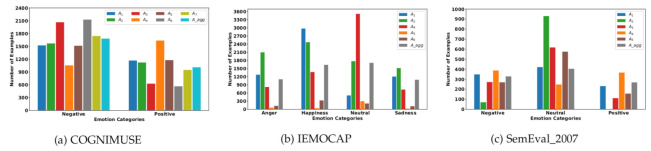
(**a**) Distributions of negative and positive examples with respect to each individual and aggregated annotations. (**b**) Represents the number of samples in each category annotated by each annotator also with aggregated annotations. (**c**) Number of positive, negative, and neutral samples annotated by multiple annotators and the aggregated annotations.

The emotional perception of each human depends upon different contextual cues, such as the situation, time, social culture, and beliefs, which makes the experience of emotion to be subjective [1,2,3]. This is also observed when we explore annotations on emotion perception or emotion experience, we can empirically observe this subjectivity. For example, Figure 1 shows the histogram (total count) of annotated emotion classes for all three datasets with respect to each individual annotator. We can observe how the histogram of each individual annotator is different from any other annotator in the same dataset. This shows the annotator’s subjectiveness when annotating any emotional dataset.

In supervised machine learning, the systems are trained based on human-annotated data. Concretely, a number of annotators manually annotate each data sample into predefined categories (dependent upon the task). Then, the common practice is to aggregate the different annotations, for example, by computing average scores or majority voting. These aggregated annotations are referred to as gold/hard/true labels whereas the different annotations are called soft/subjective labels. Usually, these aggregated labels are the ones that are released publicly with the datasets, and the researchers train and test their models on these aggregated labels. The goal of using these aggregated labels is to suppress or mitigate the noise that might come from subjective judgements. However, some tasks, including those related to affect perception, are highly subjective. For example, different people might experience different emotions when reading a piece of text or when watching a video, and all these different emotional experiences should be considered true labels.

In this paper, we address the emotion subjectivity problem. Concretely, we propose a generic Multi-task Learning approach (MT) to model affect-related tasks involving different annotators in contrast to the generic, and more traditional, Single-task approach (ST), where only the aggregated annotations are modeled. The generic ST and MT approaches are described in Section 2. Concretely, we consider an arbitrary input that can be represented by *M* different modalities. Then, the goal is to model the subjective task. In this case, we assume each sample has been labeled by multiple annotators, which might have different opinions, i.e., which might have produced different labels for the same sample. We also give a description of the three datasets that will be used in the experiments: COGNIMUSE [4], IEMOCAP [5], and SemEval_2007 [6].

Section 3 shows the experimental results that compare the ST and MT approaches on different datasets. Concretely, we used the three public datasets we could find that were providing the separate annotations of each individual annotator, instead of just providing the aggregate annotation (e.g., average or majority voting). Per each of the three datasets used (COGNIMUSE, IEMOCAP, and SemEval_2007), we offer an annotator agreement analysis showing the subjective nature of the annotations of each dataset; a description of the architecture backbone for our ST and MT modeling approaches; and the performed experiments. In the experiments, we compare the ST and MT modeling approaches. Additionally, per each dataset, we also compare the results obtained with our ST and MT approaches with the corresponding state-of-the-art baseline.

The obtained results show a similar trend for all the datasets: the MT approach outperforms the ST approach; and the MT approach outperforms the corresponding state-of-the-art. For example, in COGNIMUSE, we obtain 90.40% classification accuracy on the aggregated label with our MT, while the state-of-the-art baseline model was obtaining 83.2%. For MT classification in IEMOCAP, again the MT showed better performance in modeling subjective and aggregated emotions: MT achieves 61.51% against the 56.75%-ST and the 61.48%-Baseline. Finally, for the SemEval_2007 dataset, we observed again a similar pattern in modeling subjective emotion and aggregated emotions using MT. It scored 59.10%, whereas the Single-Task and the baseline received 57.40% and 55.10%, respectively, for aggregated emotions.

This work is based on a previous study on modeling the emotions evoked by movies [7]. In [7], the authors proposed a specific bi-modal emotion modeling technique that jointly modeled the emotions experienced by different people while watching a movie in a Multi-Task manner. This Multi-Task approach showed better results than modeling each person separately with a Single-Task approach [7]. While this previous study focused on the emotions evoked by movies combining visual and text modality, this work extends the modeling approach to a more generic formulation, and presents further experiments in more affect-related datasets. Furthermore, this work also expands the annotators’ subjectivity problem in two major ways: (i) the dimensions in which the machine learning researchers consider annotators’ subjectivity, and (ii) the limitations of different proposed approaches that deal with annotators’ subjectivity.

Overall, our experiments show that jointly modeling multiple single annotators, along with the aggregated annotator, provides better results than modeling the individual and aggregated labels with a Single-Task (ST) approach. We hope that our results will encourage the community to release individual annotations on those future data collection efforts involving subjective tasks.

### 1.1. Related Work

Generally, emotion recognition using multimodalities has been an attractive research area in Affective Computing [8,9,10]. Researchers fuse different modalities such as visual, audio, textual, and Physiological signals to recognize what emotions are experienced by the user to get a high level of accuracy [11]. However, these methods do not explicitly take into account the fact that emotions are subjective.

Some previous works have addressed the problem of subjectivity in Affective Computing, and also the problem of personalization, which is closely related to the subjectivity problem. For example, Zhao et al. [12] addressed the subjectivity problem in the context of social networks. Therefore, cues such as social context, location, or temporal evolution are considered to predict the personalized emotion perceived by a user. However, our proposed approach does not use any metadata on the annotator’s context. On the contrary, our approach attempts to learn the common patterns from the input data directly. In addition, Shahabinejad et al. [13] addressed the personalization problem, which is a problem that is closely related to the subjectivity problem. More concretely, Shahabinejad et al. [13] assumed that there is only one ground-truth label for each input sample, whereas we are addressing the problem of modeling the possible disagreement among the different annotators. These are two complementary approaches. Our work, which addresses the subjectivity problem, could be also integrated into any personalization approach. Notice that the personalization approach is tackled from the content side (face features from the images) instead of the annotation side.

In general, the affect-related recognition models are trained on single-label datasets [14,15], where the single-label is obtained by aggregating the labels of different annotators. Concretely, the single label is the outcome of the majority voting or averaging approaches employed on different ground truth labels from multiple annotators in the creation of the datasets. For example, the practice of training the models using just the aggregated labels is common in the problem of recognizing emotions evoked by audio-visual content.

More generally, just considering aggregated labels is also a common practice for other affect-related tasks such as Image Sentiment Analysis [16], Text Sentiment Analysis [17], or Expressed Emotion Recognition [18]. We also find a large variety of elaborated methods for recognizing the emotions evoked by movies [19,20]. Researchers have used different input modalities including audiovisual data, text information, and recently physiological signals. For example, Thao et al. [21] used audiovisual data for predicting affective responses of viewers watching a movie. Lee et al. [22] concatenated the visual and EEG features to understand the emotional state of viewers while watching a movie. Nguyen et al. [23] proposed a multimodal approach that uses audiovisual and text data to understand the emotions evoked by movies. Lee et al. [24] proposed a CNN (Convolutional Neural Network) that used Photoplethysmogram features to detect emotional content. Ahuja et al. [25] used two types of text features including TF-IDF word level and N-Gram for sentiment classification. Kush et al. [26] proposed an attention-based CNN model that can focus on the words that contribute more in the text emotion classification. While all these works are very inspiring in terms of modeling approaches, we notice that all of them focus on modeling just the aggregated annotations.

One of the reasons why researchers focus on the aggregated annotations in subjective tasks is because most of the public datasets just provide the aggregated annotations [16,17,18]. Fortunately, the community has recently started to release raw annotations and the demographic information of annotators [27,28]. However, these datasets only hold the text modality. In contrast, COGNIMUSE [4] and IEMOCAP [5] consist of audio, video, and text information. These two datasets have been annotated by multiple annotators and the raw annotations of each annotator are available.

To incorporate subjectivity in an algorithm, researchers usually make one of the following two assumptions: (i) *Subjectivity as Noise*, and (ii) *Subjectivity as Information*. In the first approach, researchers use soft labels in finding the underlying gold labels using different techniques such as the reliability of the annotator [29,30,31,32,33,34]. In the second approach, researchers are interested in modeling subjectivity. Related approaches use soft labels in modeling the tasks. For example, in [35,36], the authors proposed a model to predict the emotions of each annotator.

Our work belongs to the *Subjectivity as Information* perspective. We use subjective perception (soft labels) and aggregated perception (hard labels) together in a joint manner. The main idea is to preserve the subjective perception (i.e., inter-annotator disagreement) of each annotator and also get the benefits of aggregated perception (i.e., inter-annotator agreement) in affect recognition tasks. Our hypothesis is that the common patterns between each subjective and aggregated perception will boost the learning accuracies for both the individual subjective labels and the aggregated label. The two following subsections provide a brief literature review for the *Subjectivity as Noise* and the *Subjectivity as Information* perspectives.

#### 1.1.1. Subjectivity as Noise

The idea of the *Subjectivity as noise* approach is to design methods that can find the true ground truth by using the multiple noisy annotations. For example, Raykar et al. [30] proposed an approach that uses multiple annotations in their experiments. The main objective is to find the true gold annotation using multiple noisy annotations. The authors assumed that the disagreeing annotations hold the noise. The authors used prior information for each annotator to capture the skills. The work is related to finding the quality of the data annotation. This work assumes that there is a single gold standard annotation that exists behind multiple noisy annotations, and this is why the approach produces a single output.

Another technique in the series of findings on the reliability of the data annotations is presented by Yan et al. [31]. The previously mentioned approach [30] assumed that the annotations are dependent on the annotator’s expertise. Whereas, in this paper, the authors assume that the expertise level is dependent on the data sample that each annotator observes. With this assumption, the authors model the error rate of each annotator as dependent on the data sample. Morales-Álvarez et al. [32] proposed a Gaussian Processes (GP)-based approach that handles multiple annotations without knowing their expertise level. The objective of this approach is similar to what we discussed in previously presented approaches, i.e., using multiple annotations to obtain a single true annotation. After fully converging, the proposed model gives less importance to those annotators who produce noisy annotations. Cohn et al. [33] also proposed a Gaussian Processes (GP)-based multitask learning to model the subjectivity of different annotators based on their level of expertise and reliability. This approach is different from our Multi-Task (MT) in the following ways. Firstly, the prior for each annotator’s function must be correlated to each other whereas, in our approach, there is no such type limitation on setting the priors of each annotator’s function. Secondly, the proposed [33] multitask algorithm recognizes that it is very rare that the annotators are independent of one another, i.e., often annotations are dependent on each other.

Rodrigues et al. [34] also considered that each annotator has a different level of expertise. The authors proposed a crowd layer on the top of the output layer called the bottleneck layer. This crowd layer has multiple outputs, each output belongs to each individual annotator. The crowd layer learns the annotators’ behavior and adjusts the annotator bias according to the labels assigned to each annotator. The adjusted gradient from each annotator is then passed to the previous bottleneck layer. This layer aggregates the multiple gradients that are coming from *N* annotators and backpropagates down to the network. This crowd layer is only used for the training time, i.e., once the network is fully trained, then it is removed. In the testing phase, the bottleneck layer acts as an output of the network. Since the output layer is converged based on the aggregation of multiple gradients, it is not clear that the output represents each annotator and, hence, there is a loss on the subjectivity.

In summary, most of the studies that considered subjectivity as noise have been done in the domain of crowdsourcing, where we have hundreds or thousands of annotators for annotating a single data sample. Researchers use subjective labels to find the reliability of the annotators and based on the reliability score, they give weight to a particular annotation in order to find the underlying gold label. The proposed approaches were evaluated on tasks that are less subjective in nature than affect-related tasks, such as Breast malignancy [31], Quality Estimated of translated sentences [37], Image classification (cats and dogs [38]/multi-category such as highways, streets, forests, etc. [39]), and human activity recognition (such as walking, standing, etc.) [40].

#### 1.1.2. Subjectivity as Information

The *Subjectivity as Information* approach assumes that each single annotation contains meaningful information worth to pay attention to. Under this perspective, Fornaciari et al. [41] proposed a multi-task model that learns from multiple annotations. The multi-task model is built on the top of the single-task, where the single-task predicts the standard single output, and this single output is treated as the distribution of labels. Then, an additional task is added on top of it called an auxiliary task. The aim of this additional task is to predict the soft label distributions with respect to each annotator. The training of both tasks is done in a joint manner. Two different losses are computed: one for the main task (predicting gold labels) and one for the auxiliary task (predicting soft labels). The proposed multi-task was evaluated on two different tasks: POS (Part-Of-Speech) tagging and morphological stemming. In this case, it is assumed that there exists a unique ground truth, which is not necessarily the case in affect-related tasks, where perceptions or experiences can be genuinely different.

In the context of addressing emotion subjectivity, Fayek et al. [35] trained multiple ensembles Deep Neural Networks (DNNs), each representing a single annotator. The multiple ensemble DNNs were trained against two different types of labels: (i) *Hard labels*, and (ii) *Soft Labels*. In [35], the authors referred to each individual annotator’s annotations as hard labels. Each ensemble DNN was trained against the ground truth that belongs to a single annotator. To get soft labels from each annotator’s hard labels, the author used an encoding scheme proposed in [42]. Later, they used two different techniques to combine the outputs of each ensemble, one is called Geometric Mean, and the other is called Unweighted Majority. The model was tested on the IEMOCAP dataset. The approach has a major limitation: the ensemble approach was only tested using three annotators without mentioning their ids. The categorical label was annotated by six different annotators for the IEMOCAP dataset and it is unclear which three annotators were used out of six annotators [5]. Secondly, the author’s implementation is not publicly available. Third, the authors did not provide each ensemble DNN performance.

In our case, we are interested in modeling the subjective as well as the aggregated labels. In this context, Chou et al. [36] proposed an approach that uses the aggregated annotations (hard label) and the distribution of annotations (soft label) simultaneously in a joint manner to address emotional subjectivity. The learning consists of multiple models, i.e., five models for each individual annotator, and a combination of two previous models ([43,44]). Later, they concatenate all the outputs and use Softmax for the final predictions. The approach is based on the concatenation of two different types of subjective perception: (i) modeling subjective perception using original soft labels, i.e., labels that are annotated by each individual annotator, and (ii) generating soft labels using the approach proposed by Ando et al. [43] and then modeling the subjective perception. The authors did not provide clarity about what type of soft labels is considered to address subjectivity in general. For example, original soft labels that are directly coming from annotators or soft labels that are obtained from any encoding scheme or a combination of both. This limits the possibility of considering this approach as a general approach. The approach is evaluated in a single dataset (IEMOCAP) on the audio modality and it achieved state-of-the-art results in modeling emotions for individual and aggregated annotations on the IEMOCAP dataset. For this reason, we compare our proposed Multi-task (MT) model with this approach for IEMOCAP in Section 3. However, we consider that this approach is not general enough to be used in other datasets, partially because the model itself is based on two previous models that have been explicitly designed for IEMOCAP.

## 2. Materials and Methods

This section presents the generic Single-Task (ST) and Multi-Task (MT) modeling approaches, and describes the datasets used in our experiments as well as the backbone architectures used per each dataset and modality. The last part of the section describes the evaluation protocol and metrics used in our experiments.

### 2.1. Generic Single-Task (ST) and Multi-Task (MT) Modeling Approaches

In this paper, we propose a new perspective on modeling affect-related tasks, which are highly subjective. Thus, the scenario considered in this work is the following: given an input sample, there are multiple annotators ($A_{1},...,A_{N}$) which provide a label for the input sample according to an affect-related task (e.g., emotion evoked by the sample, sample’s sentiment, etc.). As previously discussed, these types of affect-related tasks are subjective. Therefore, the labels provided by different annotators on the same sample would be different. In order to analyze how annotator-level labels can be leveraged for modeling the aggregated annotator label, we compare two types of CNN architectures: (i) a Single-Task (ST) architecture, where each annotator is considered independently, and (ii) a Multi-Task (MT) architecture, where all annotators are considered jointly.

#### 2.1.1. Single-Task (ST) Architecture

The Single-Task (ST) architecture produces a single label as the output for each input sample. This single label may come from either a specific annotator or from an aggregate annotator after applying majority voting, average, or any other aggregated annotation. This architecture allows us to train a different specific model for each annotator but it does not exploit some common patterns that the different models may find.

**Figure 2:**
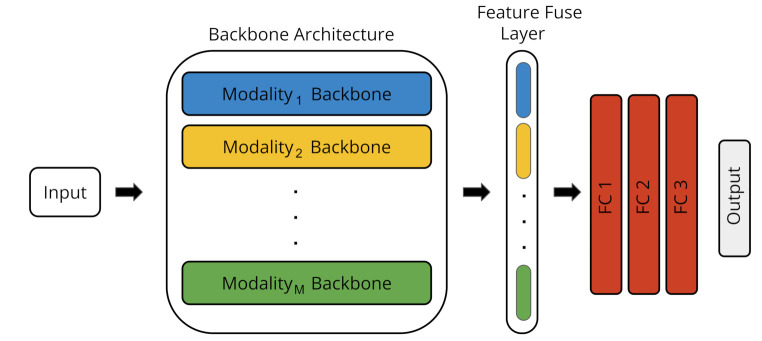
Architecture of the Single-Task (ST) model. It consists of feature extractors for each modality, a feature fusion layer, and a fully-connected block.

Our ST architecture is illustrated in Figure 2. The architecture consists of three different blocks: (i) the backbone architecture, (ii) the feature fusion layer, and (iii) the fully connected block.

The backbone architecture consists of as many branches as the number of modalities of the input data which will be considered for the emotion recognition model. Examples of modalities are visual modality, text modality, or audio modality.

Once the features are obtained for each modality from the backbone architecture, the features are fused in the feature fusion layer by concatenation, resulting in a multimodal representation of the data.

Finally, the fully connected block consists of a sequence of fully connected layers. The loss function used in the architecture would depend on the inference task (e.g., classification, regression, etc.).

**Implementation details.** In the experiments of this paper, we use the following implementation details for the training. The fully connected block is composed of three fully connected layers with 1024, 512, and 256 units, respectively. We use the Adam/Gradient Descent optimizer [45,46] for training, with a learning rate $10^{-3}$. We use Lasso regularization [47] to avoid overfitting. Regarding the loss function, all the tasks tested in this work are classification tasks. Thus, we use cross-entropy loss.

#### 2.1.2. Multi-Task (MT) Architecture

**Figure 3:**
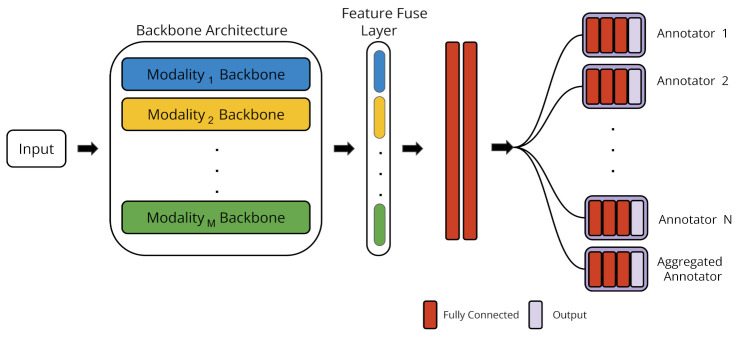
Architecture of the Multi-Task (MT) model. This architecture is applied to all annotators in a joint manner by sharing two fully-connected layers and a dedicated fully-connected block for each annotator.

The Multi-Task (MT) architecture produces multiple output labels per each input sample: one per annotator. The MT architecture is illustrated in Figure 3. Similarly to the ST architecture, the MT architecture also has three different blocks: the backbone architecture, the feature fusion layer, and the fully connected block. The backbone architecture and the feature fusion layer are the same in both ST and MT architectures. The difference between both architectures lies in the fully connected block.

The MT architecture has two fully connected layers after the feature fusion layer, which are shared among all the annotators. These two shared layers allow the model to learn the common patterns found in the data. After that, a separate fully-connected block is considered for each annotator to learn their specific annotations. Each separate block consists of 3 fully connected layers. As in the ST architecture, the loss function is selected according to the nature of the target task.

Notice that, besides each individual annotator, a separate block is also considered for the aggregated annotator. Therefore, the model does not only learn the specific patterns for each annotator, but also addresses the task of modeling the aggregated label.

**Implementation details.** In our experiments, we use the following implementation details. The two shared fully connected layers have 2048 and 1024 units, respectively. The three fully connected layers of the annotator-specific separate block have 512, 256, and 128 units, respectively. As in the ST architecture, the sigmoid/softmax function is applied to the output layer of each separate block to get the final prediction for that specific annotator. The weights of the shared backbone block and the two fully-connected layers are updated in every training batch whereas the weights of the fully-connected block from a separate branch are only updated when the batch contains annotations of that specific annotator. As in the ST architecture, we use the Adam/Gradient Descent optimizer with a learning rate $10^{-3}$ and we also use Lasso regularization to avoid overfitting.

### 2.2. Datasets

We compare the ST and the MT modeling approaches in 3 affective datasets/tasks: COGNIMUSE [4]—emotions evoked by movies, IEMOCAP [5]—affect recognition in audio, and SemEval_2007 [6]—text sentiment recognition.

The COGNIMUSE dataset is a multimodal video dataset [4]. The dataset is generated for multiple tasks such as audio-visual and semantic saliency, audio-visual events, action detection, cross-media relations, and emotion recognition. The dataset includes movies and travel documentaries with human annotations.

For video emotional understanding, the dataset [4] included 7 Hollywood movies: “Chicago” (CHI), “Finding Nemo” (FNE), “A Beautiful Mind” (BMI), “Crash” (CRA), “Gladiator” (GLA), “Lord of the Rings— the Return of the King” (LOR), “The Departed” (DEP) and considered 30 min of video content from the start of the movie. The emotional contents in the movies are represented in arousal and valence dimensions, where arousal encodes the viewer’s excitement and valence represents how positive or negative is the emotion evoked by the clip. The viewers annotate each movie clip into many small segments. The length of a single segment is 40 ms and 7 different viewers annotate each frame in continuous values from −1 to +1.

The IEMOCAP [5] is an acted, multi-speaker dyadic dataset. A total of 10 different actors (5 male, 5 female) took part in recording their face motion, head movement, speech, and visual data. The actors played their roles in two different settings: scripted and spontaneous. The recordings were done in sessions and there are a total of 5 sessions in the dataset. After recording the sessions, the dialogues in each session were segmented into utterances. Each utterance was annotated into 9 categorical (anger, happiness, excitement, sadness, frustration, fear, surprise, other and neutral state) and 3-dimensional (valence, activation, dominance) labels. Six annotators took part to classify the emotional content of utterances into categorical emotion dimensions. Each utterance was annotated by at least three annotators. On the other hand, for the continuous dimension, two annotators annotated the whole dataset. Only annotators $A_{1}$, $A_{2}$, $A_{4}$, $A_{5}$, $A_{6}$, and aggregated $A_{agg}$ (majority voting) hold enough annotations which can be used for modeling their emotional perception. The emotions classes used in our experiments are the same as the ones considered in [36]: Anger, Happiness, Neutral, and Sadness. Furthermore, as done in [36], all samples originally annotated as Excitement are also included in the Happiness category.

The dataset SemEval_2007 [6] was developed to evaluate the participating systems in order to classify the emotions in news headlines. The dataset consists of 1000 news headlines that were taken from different news channels, including CNN, BBC and Google News, and the New York Times newspaper. The news headlines were annotated in 6 categorical emotions (Anger, Disgust, Fear, Joy, Sadness, Surprise) and 1 continuous dimension, i.e., Valence. The range of the categorial emotion was set between 0 and 100, where 0 represents No emotion and 100 represents the maximum intensity of the emotion category. On the other hand, the Valence dimension was set between −100 and 100, where −100 represents Very Negative, 0 for Neutral, and 100 for Very Positive. The dataset is annotated by 5 different annotators. To model both individual and aggregated annotations, we added aggregated annotations (average of all annotations) in the dataset. We only considered the Valence dimension for coherence with the experiments done on COGNIMUSE.

### 2.3. Backbone Architecture Details

Per each dataset and modality, we use specific backbone architectures that are based on the corresponding architectures that obtain state-of-the-art results in each dataset. Below, we provide all the details.

#### 2.3.1. Backbone Architecture for COGNIMUSE

We used Visual, Text, and Audio modalities for the recognition of evoked emotions using the COGNIMUSE dataset. Below, we describe the feature extraction backbone for each modality.

**Visual Modality.** The visual backbone consists of a pre-trained RGB-I3D model [48] that was trained on the Kinetics-400 Dataset. The model is inspired by the state-of-the-art model named Inception-V1. To build the RGB-I3D model, all the convolutional and pooling operations in the hidden layers of Inception-V1 were converted from dimension 2 into dimension 3. This third dimension helps the model in learning the temporal patterns of the videos. Each convolutional layer is followed by max-pooling, batch normalization, ReLU activation, and a softmax function. The model provides multiple endpoints for collecting the features of the given video. In our experimental settings, we collected video features from the last endpoint of the model called “Mixed-5c”. We processed each video clip with a batch of 16 consecutive frames and set a stride of 8 frames. Later, we used global average-pooling to get the features. Finally, we performed max-pooling across the temporal domain.

**Text Modality.** The textual features are learned using a word-embedding matrix [49]. It is a technique in which the words that have the same meaning have a similar representation. We first convert each word in a sentence into a d−dimensional vector. Through this, sentences can be represented as vectors of numerical values. In our dataset, the maximum number of words in any sentence is 18. All the sentences that are shorter than 18 words are padded with a particular word at the end of the sentence. This procedure is done to get the same length of each sentence in the dataset. Later, we used Convolutional Neural Network (CNN) to learn the feature patterns of the textual data. The network consists of a sequence of two pairs of convolution layers with max-pooling, two hidden layers, and an output layer. All the hyperparameters of the CNN were initialized randomly and trained with the labeled data.

**Audio Modality.** For the audio modality, we use the pre-trained VGGish [50] to get audio features. VGGish is a modified version of VGG (configuration E) [51]. It is trained on the large-scale audio events dataset called AudioSet [52] having 632 audio classes. In our experiments, we use the features of the last convolution layer that has 512 kernels. Before feeding raw audio into the model, some preprocessing steps are done. The audio sequence of each corresponding clip is first divided into *n* number of frames having the length of 960 ms, where *n* represents the length of the clip in seconds. After getting the frames, the next step is to extract the spectral information of each frame, i.e., how much energy the windowed signal contains at different frequency bands. The Short-Time Fourier Transform (STFT) is used for extracting the spectral information with a 25 ms window length and a 10 ms window shift. STFT transforms each 960 ms frame into a 64 Mel-spaced frequency vector, and the magnitude of each bin is log-transformed. This gives the 2D log-Mel-spectrogram patch of 96∗64 bins. Each audio clip results in a n∗96∗64 tensor. To obtain the most important frequencies of the clip, we then apply max-pooling across the temporal domain.

#### 2.3.2. Backbone Architecture for IEMOCAP

To compare with Chou et al. [36], which is the model that obtains state-of-the-art results on IEMOCAP, we only used audio modality in our experiments. For the audio feature of each utterance, we followed the same approach as we used in the COGNIMUSE dataset (see Section 2.3.1). In the audio preprocessing step, the audio sequence is first divided into *n* number of frames having the length of 960 ms. In the IEMOCAP dataset, few utterances were shorter than 960 ms; so, to adjust this, we used silence padding after the utterance.

#### 2.3.3. Backbone Architecture for SemEval_2007

All the news headlines from SemEval_2007 dataset are textual. For text feature representations, we used a pre-trained language representation model called BERT [53]. It is based on the Transformers [54], which obtained state-of-the-art results on a wide range of Natural Language Processing (NLP) tasks. BERT is available in many different configurations. From those, we selected the Uncased-BERT-Base configuration for our experiments. We considered the output of the PooledOutput layer, which generates a 768-dimensional vector for each news headline for further classification.

### 2.4. Evaluation Protocol

In this section, we detail how we evaluate our approach with respect to previous modeling approaches on each of the datasets. In particular, we test the Single-Task (ST) and Multi-Task (MT) approaches and we compare them with the state-of-the-art approaches related to each particular dataset. For the COGNIMUSE dataset, we could not find any work that uses subjective annotations and, to the best of our knowledge, we are the first ones who used the subjective annotations of the COGNIMUSE dataset. With this limitation, we could compare our Single-Task (ST) and Multi-task (MT) approaches with previous works only for aggregated annotations. The multimodal approach of Nguyen et al. [23] has the state-of-the-art results in modeling a single emotion dimension (Valence) using aggregated annotations. Besides comparing our approaches with state-of-the-art for aggregated annotations, we also present the comparison between our ST and MT, to show how the Multi-Task approach surpasses the Single-Task for each subjective perception.

Similarly, we could not find any work that uses the subjective annotations of the SemEval_2007 dataset. This is why we compare our ST and MT approaches only for aggregated annotations with the state-of-the-art approach. Strapparava et al. [6] presented a model named CLaC that has achieved state-of-the-art results in modeling 3 emotion classes (Negative, Neutral, and Positive) using text modality. For the subjective perception, we followed the same approach as we adopted for the COGNIMUSE dataset.

For the IEMOCAP dataset, the approach of Chou et al. [36] has achieved the state-of-the-art results in modeling 4 emotion classes (Anger, Happiness, Neutral, and Sadness) using only the audio modality. The authors used both subjective and aggregated annotations in their experiments. We compare our Single-Task (ST) and Multi-Task (MT) with [36] for modeling subjective and aggregated emotional perception.

In terms of performance metrics, we used two different types of evaluation metric: (i) the overall accuracy (with respect to all the possible classes), and (ii) the Unweighted Average Recall (UAR). The reason behind this is to do a fair comparison with the state-of-the-art. Nguyen et al. [23] and Strapparava et al. [6] used the overall accuracy for COGNIMUSE and SemEval_2007 datasets, respectively, whereas the approach of Chou et al. [36] used UAR to evaluate the classification performance of the model for IEMOCAP dataset.

## 3. Results

This section presents the results on 3 public affect-related datasets that provide the individual annotations of multiple annotators: COGNIMUSE [4], IEMOCAP [5], and SemEval-2007 [6]. These three datasets are affect-related datasets labeled on subjective tasks. Notice that the subjectivity of the tasks are illustrated in Figure 1, which shows per each dataset the total number of samples per class provided by each annotator. We can observe that for all the datasets, these counts vary across annotators, which shows the diversity of opinions.

For each dataset, we first analyze the annotator agreement, which empirically illustrates the subjectivity of the tasks. For this, we compute the Cohen Kappa statistic to measure the agreement between every pair of annotators, including the aggregated annotator. Then, we show the results obtained with the ST and the MT, and compare them with the state-of-the-art model of each dataset. We also provide some qualitative results.

### 3.1. Results on the COGNIMUSE Dataset

In our experiments, we only focus on the Valence dimension in order to compare our proposed ST and MT architectures with a baseline [23]. The continuous Valence values are converted into binary labels by considering the 0 value as a threshold, being considered as positive when the valence values are greater than 0 and negative otherwise.

#### 3.1.1. Annotator Agreement Analysis

**Figure 4:**
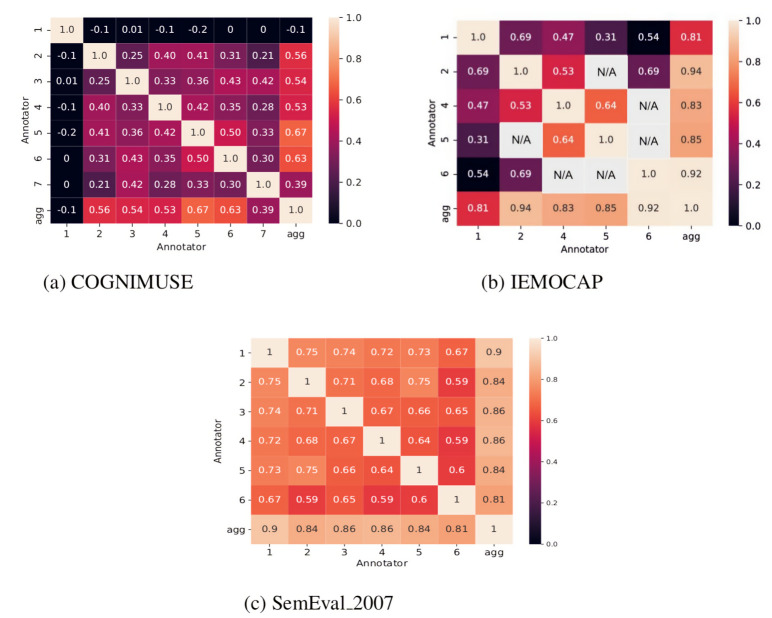
Pairwise inter-annotator Cohen’s kappa of COGNIMUSE, IEMOCAP, and SemEval_2007 datasets.

The authors of the COGNIMUSE dataset [4] provided an analysis of the inter-annotator agreement, obtaining a Pearson correlation value of 0.29 for the valence values. This low value for agreement shows that the individual emotional experience is highly subjective. We extended the inter-annotator agreement analysis and measured the pairwise inter-annotator Cohen’s kappa values (Figure 4). This is done to understand the emotional subjectivity between each individual as well as with the aggregated emotions. We found that there is a low correlation between each pair of annotators, but a higher correlation between every single annotator with respect to the aggregated annotations (average of all annotations). This higher correlation between every single annotator and the aggregated annotator motivates the idea that a multitask learning approach could leverage the patterns learned from each individual annotator for the prediction of the aggregated annotator.

#### 3.1.2. Quantitative Results

Firstly, we trained our Single-Task (ST) and Multi-Task (MT) models on cross-validation folds with 5 movies for training, 1 movie for validation, and 1 for testing. We ensure that each movie should be in the test set. In comparison with a previous study [7], the audio modality has been also added to the backbone architecture besides the visual and text modalities. The Multi-Task approach showed better generalization for each annotator. On average, MT has achieved 4.6 points higher accuracy as compared to the ST for all individual annotators. Furthermore, for the aggregated annotator, the improvement is even more significant, i.e., 8.78 points (see Table 1). This is strong evidence that MT learning takes advantage of all the annotators in generalization. These experiments could not be reproduced with the Nguyen et al. [23] approach since the code is not publicly available.

Secondly, the original split of the dataset used in [23] (BMI, CHI, FNE, GLA and LOR in training, CRA and DEP in test) was also considered in order to be able to compare our proposed MT architecture with that of Nguyen et al. [23]. Unfortunately, we could not compare our MT with [23] following the cross-validation protocol because the authors of [23] did not release their code. Furthermore, notice that the Nguyen et al. [23] approach was only trained on aggregated annotations; therefore, it is not possible to make a comparison for each individual annotator. Therefore, we only present the result obtained by aggregated annotators when using our MT approach (see Table 2). The MT learning performed significantly better and achieved 7.2 points higher accuracy than the Nguyen et al. [23] approach.

A deeper analysis of the results on the COGNIMUSE dataset can be found in a previous work [7]. This analysis includes the results for each partition considered in the cross-validation evaluation as well as an ablation study of the different modalities.

#### 3.1.3. Qualitative Analysis

**Figure 5:**
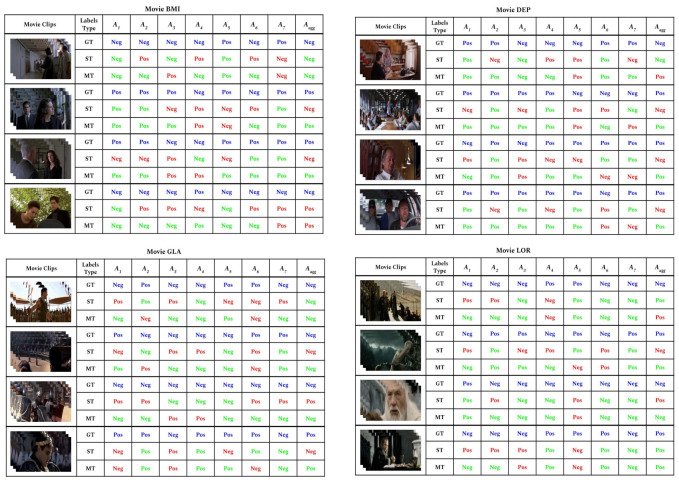
Qualitative results for some randomly selected segments of 4 different movies from COGNIMUSE. The figure shows the ground truth annotation per each annotator, including aggregated annotator, and compares the predictions of Single-Task (ST) and Multi-Task (MT) models with the Ground Truth (GT). Blue is for GT, Green is for a correct prediction, and Red is for incorrect prediction.

In this section, we present the qualitative analysis of our Single-Task (ST) and Multi-Task (MT) approaches. We randomly selected a few samples from the dataset and compare their ground truth labels with the predictions of our ST and MT models. The results are shown in Figure 5, where the ground truth (GT) of each movie with respect to each individual annotator is compared with the predictions of our Single-Task (ST) and Multi-Task (MT). We can see how MT is able of capturing the differences among the different annotators and the aggregated annotation more often than ST.

### 3.2. Results on the IEMOCAP Dataset

#### 3.2.1. Annotator Agreement Analysis

In [5], the provided inter-annotator agreement is for the entire dataset (i.e., Fleiss’ kappa = 0.48). In our experiments, we modeled each individual and the aggregated annotator. This is why the pairwise inter-annotator agreement is worth understanding each annotator’s emotional perception (see Figure 4). The “NA” means that there is no sample annotated by these pairs of annotators. The low inter-annotator Cohen’s kappa shows that emotions are highly subjective. On the other hand, the high Cohen’s kappa between each individual and the aggregated annotators is interpreted as the loss of emotion subjectivity and hence proves our hypothesis.

#### 3.2.2. Quantitative Results

We tested our Single-Task (ST) and Multi-Task (MT) learning approaches using the leave-one-session-out cross-validation fold. As compared to the Baseline [36] in which 4 sessions were used for training and 1 for testing, we used 3 sessions for training, 1 for validation, and 1 for testing. We ensure each session should appear in the validation and the test set. Whereas we were addressing a binary classification problem in COGNIMUSE dataset, a multi-class classification problem with four different emotion categories is tackled on IEMOCAP. The accuracies are reported for each individual and the aggregated annotators with respect to all the possible classes.

As it can be seen in Table 3, we found the same pattern for each single and the aggregated annotators as we observed when using the COGNIMUSE dataset. On average, the MT has achieved 8.27 points higher UAR (Unweighted Average Recall) for each individual annotator and 4.76 points higher UAR for the aggregated annotator as compared to the ST. When we compared the MT results with the Chou et al. approach [36], the MT learning achieved significant improvement in modeling annotators 1, 4, 5, and 6, where the point differences are 6.09, 10.93, 17.88, and 3.55, respectively. For the aggregated annotator, the MT performed slightly better than the Chou et al. [36] approach. As compared to Chou et al. [36], on average, our MT approach has increased the classification performance with an increment of 6.41, including the individual and the aggregated emotional perception.

The reason behind this significant improvement in Annotator 4 and Annotator 5 is due to the Shared Fully-Connected Block in Multi-Task (MT) learning, which helps to improve the performance of each individual and also the aggregated annotator. In Table 4, we can clearly observe the differences in the number of samples per emotion category with respect to each individual annotator and the aggregated annotator. Annotator 5 has the lowest number of samples per each emotion category. However, thanks to the Shared Fully-Connected Block, the separate block of Annotator 5 improves the performance. The results prove that the Shared Fully-Connected Block learns the patterns that are common in each individual and the aggregated annotator and helps in the classification performance of each individual and the aggregated annotator. Our results also show that the Shared Fully-Connected Block in our proposed Multi-Task (MT) approach can deal with imbalanced datasets and helps in improving the classification performance (see Table 5). Overall, the proposed MT approach again showed that it is best suited in subjective learning from single and aggregated annotators.

#### 3.2.3. Qualitative Analysis

In Table 6, we present a few utterances that were randomly selected from 5 different sessions of the IEMOCAP dataset. Each utterance was annotated by 3 different annotators. The annotations from all 3 annotators and the aggregate annotators are compared with our ST and MT approaches. Again, we can see how the MT approach outperforms the ST approach more often.

### 3.3. Results on SemEval_2007 Dataset

#### 3.3.1. Annotator Agreement Analysis

In SemEval_2007 [6], Pearson correlation was measured to understand the inter-annotator agreement for the entire dataset. The inter-annotator agreement for the Valence dimension is 0.78. The agreement measure is a bit high but still holds a subjective nature. Since we are modeling each single and aggregated emotion, pairwise inter-annotator agreement analysis gives a better understanding (see Figure 4). We found the same pattern as we found in COGNIMUSE and IEMOCAP datasets, i.e., the low agreement between every single annotator and a high agreement between each annotator and the aggregated annotator, which supports our multi-task learning hypothesis.

#### 3.3.2. Quantitative Results

We tested our ST and MT approaches using 10-fold cross-validation. We divided the 1000 news headlines into 10 different folds having the same number of news headlines in them. We selected 7 folds for training, 2 for validation, and 1 for testing. We ensured that each fold must be in a test set. The model accuracy is calculated with respect to all 3 classes considered for the Valence dimension: Negative, Neutral, and Positive. Consecutively, we observed the same learning pattern of MT as we observed for COGNIMUSE and IEMOCAP. As it can been seen in Table 7, the experiment results show that the MT learning outperformed the ST approach. On average, MT achieved 1.96 points higher accuracy than ST for every single annotator. For the aggregated annotator, MT is also 1.70 points better than ST in terms of accuracy. Table 8 compares the results with the baseline approach [6]. It is worth noting that our models were trained and validated on the same dataset using a cross-validation approach, whereas the baseline was trained on another dataset.

#### 3.3.3. Qualitative Analysis

For SemEval_2007 data, we randomly selected 5 sentences and compare their ground truth (GT) with the predictions of Single-Task (ST) and Multi-Task (MT) models with respect to each individual annotator. We can see the same trend we have observed with the other two datasets: the MT approach is more often accurate with respect to each individual annotator and the aggregated annotation (see Table 9).

## 4. Conclusions

This paper addresses the problem of subjectivity in supervised Machine Learning in the context of affect recognition. In general, the problem of subjectivity has been addressed broadly from two main perspectives: “Subjectivity as noise” and “Subjectivity as information”. Our work is contextualized in the “Subjectivity as information” perspective, assuming that affect recognition tasks are subjective in general, and the different opinions of different annotators are entirely informative.

In this study, we test a generic Multi-Task (MT) formulation on three different affect-related datasets: COGNIMUSE, IEMOCAP, and SemEval_2007. The MT model jointly estimates the annotation of each separate annotator and the aggregated annotation, producing one output per each annotator and, additionally, one output for the aggregated annotation. We compare this generic MT architecture with the traditional Single-Task (ST) approach and also with the models that produce state-of-the-art results in each of the considered datasets. The results show that the proposed MT formulation performs systematically better in modeling the subjective and the aggregated annotations when compared to both the ST approach and the state-of-the-art models.

Overall, our results empirically show that an MT model that jointly learns individual and aggregated annotations can better learn the individual annotations and the aggregated annotations. However, the results obtained with the proposed approach can still be improved, which shows that Multi-Task (MT) is still limited to entirely model subjective annotations. First, our study is conducted on the few public datasets we could find that release the individual annotations. These datasets have a limited size and variability. In particular, the number of annotators is never larger than 7, which does not allow one to study how to deal with the subjectivity problem with a larger number of annotators. One possible way of dealing with more annotators would be to use clustering to create groups of similar annotators, and then use MT with a separate branch per each group of annotators. This would be future work that could be done with a larger dataset that includes more annotators. Furthermore, affect recognition tasks are very challenging in general since there are many contextual cues that can affect the subjectivity experience or perception of emotions [55]. In our work, we are addressing the subjectivity problem in a holistic way, but further research is needed to understand with more depth the subjective judgement and to create Machine Learning approaches that can deal with the subjectivity problem in a robust manner. We expect that our study motivates the release of individual annotations in further dataset collections in the context of affect recognition. All the code and trained models resulting from this work will be released upon the publication of the paper.

## Figures and Tables

**Table 1:** ST vs. MT comparison on the COGNIMUSE dataset. Results of ST and MT when modeling single and aggregated annotators with cross-validation evaluation.

Annotator_ID	Methods	
	Single-Task (ST)	Multi-Task (MT)
$A_{1}$	65.86	**70.09**
$A_{2}$	68.50	**72.13**
$A_{3}$	66.00	**71.38**
$A_{4}$	71.08	**74.29**
$A_{5}$	78.47	**83.07**
$A_{6}$	71.15	**76.22**
$A_{7}$	67.29	**74.05**
$A_{agg}$	71.24	**80.02**
Mean	69.94	**75.15**

**Table 2:** MT comparison with state-of-the-art on the COGNIMUSE dataset. Result of Nguyen et al. [23] and MT when modeling aggregated annotator ($A_{agg}$) with the data split from [23].

Annotator_ID	Methods	
	Nguyen et al. [23]	Multi-Task (MT)
$A_{agg}$	83.2	**90.40**

**Table 3:** Result comparison of ST, MT, and the state-of-the-art using the IEMOCAP dataset. Mean UAR (Unweighted Average Recall) is obtained with cross-validation and considering four emotion categories (i.e., 4-class classification).

Annotator_ID	Methods		
	Chou et al. [36]	Single-Task (ST)	Multi-Task (MT)
$A_{1}$	50.98	51.70	**57.07**
$A_{2}$	**59.68**	51.63	58.11
$A_{4}$	48.59	53.19	**59.52**
$A_{5}$	37.62	41.04	**55.50**
$A_{6}$	45.82	40.62	**49.37**
$A_{agg}$	61.48	56.75	**61.51**
Mean	50.69	49.15	**56.84**

**Table 4:** The number of training samples per emotion category with respect to each individual and aggregated annotator of the IEMOCAP dataset.

Annotator_ID	Emotion Categories			
	Anger	Happiness	Neutral	Sadness
$A_{1}$	1271	2970	509	1203
$A_{2}$	2095	2467	1769	1514
$A_{4}$	821	1369	3511	724
$A_{5}$	61	37	297	22
$A_{6}$	125	324	212	112
$A_{agg}$	1103	1636	1708	1084

**Table 5:** ST and MT results per each emotion category using IEMOCAP.

Annotator_ID	Anger		Happiness		Neutral		Sadness	
	ST	MT	ST	MT	ST	MT	ST	MT
$A_{1}$	52.12	**55.88**	54.63	**61.50**	51.38	**56.46**	48.68	**54.45**
$A_{2}$	51.42	**57.23**	53.27	**62.48**	51.88	**56.58**	50.26	**56.15**
$A_{4}$	51.54	**58.57**	52.64	**58.87**	55.82	**63.31**	52.79	**57.34**
$A_{5}$	48.20	**62.17**	32.56	**53.99**	49.30	**57.83**	34.13	**48.07**
$A_{6}$	36.54	**48.20**	42.85	**49.20**	40.66	**46.77**	42.46	**52.89**
$A_{agg}$	56.44	**60.00**	57.59	**61.62**	57.79	**62.36**	55.20	**58.30**

**Table 6:** Qualitative results: randomly selected utterances from 5 sessions of the IEMOCAP dataset. The table has the ground truth annotations from each individual annotator, including aggregated annotators, and predictions of Single-Task (ST) and Multi-Task (MT) models. In the table, “-” means not available and “happ” stands for happiness. Blue is for GT (Ground Truth), Green is for a correct prediction, and Red is for an incorrect prediction.

Utterances	Labels Type	$A_{1}$	$A_{2}$	$A_{4}$	$A_{5}$	$A_{6}$	$A_{\mathrm{agg}}$
I don’t understand you, do I?	GT	anger	sadness	anger	-	-	anger
	ST	anger	neutral	neutral	-	-	happ
	MT	anger	sadness	neutral	-	-	anger
What of it?	GT	happ	neutral	neutral	-	-	neutral
	ST	happ	happ	happ	-	-	happ
	MT	happ	neutral	happ	-	-	neutral
Thank you dear. The same applies to you...	GT	happ	neutral	neutral	-	-	neutral
	ST	anger	happ	neutral	-	-	anger
	MT	neutral	neutral	neutral	-	-	neutral
Well there has to be something you haven’t tried.	GT	neutral	-	neutral	neutral	-	neutral
	ST	happ	-	anger	anger	-	neutral
	MT	neutral	-	anger	neutral	-	neutral
What?	GT	anger	anger	-	-	anger	anger
	ST	sadness	neutral	-	-	neutral	anger
	MT	anger	anger	-	-	anger	anger
It’s just so much.	GT	sadness	-	sadness	sadness	-	sadness
	ST	anger	-	anger	sadness	-	anger
	MT	sadness	-	sadness	sadness	-	sadness
I was coming from um the Midwest, like Iowa.	GT	happ	-	neutral	neutral	-	neutral
	ST	happ	-	happ	happ	-	neutral
	MT	happ	-	neutral	anger	-	happ

**Table 7:** ST vs. MT comparison on the SemEval_2007 dataset for individual and aggregated annotators. Mean accuracies obtained with cross-validation.

Annotator_ID	Methods	
	Single-Task (ST)	Multi-Task (MT)
$A_{1}$	58.30	**60.60**
$A_{2}$	80.50	**82.30**
$A_{3}$	66.60	**68.40**
$A_{4}$	57.70	**59.80**
$A_{5}$	58.40	**60.20**
$A_{agg}$	57.40	**59.10**
Mean	63.15	**65.06**

**Table 8:** Comparison of state-of-the-art [6], ST, and MT on the SemEval_2007 dataset, only for aggregated annotator.

Annotator_ID	Methods		
	Strapparava et al. [6]	Single-Task	Multi-Task (MT)
$A_{agg}$	55.10	57.40	**59.10**

**Table 9:** Qualitative results: randomly selected sentences from the SemEval_2007 dataset. The table has the ground truth annotations from each individual annotator, including aggregated annotators, and predictions of Single-Task (ST) and Multi-Task (MT) models. In the table, “neg” stands for negative, “neu” stands for neutral, and “pos” stands for positive. Blue is for GT (Ground Truth), Green is for a correct prediction, and Red is for incorrect prediction.

Sentences	Labels Type	$A_{1}$	$A_{2}$	$A_{3}$	$A_{4}$	$A_{5}$	$A_{\mathrm{agg}}$
Cases: when the simple solution is the right one	GT	pos	pos	neu	neu	pos	pos
	ST	neu	neu	neu	neg	pos	neg
	MT	neu	pos	neu	pos	pos	pos
Rio De Janeiro journal: drawing lines across the sand, between classes	GT	neu	neg	neg	neu	neu	neu
	ST	neu	pos	neg	pos	neg	pos
	MT	neu	neg	neg	pos	neu	neu
Passing exchange becomes political flashpoint	GT	neu	neu	neu	neu	neu	neu
	ST	pos	pos	neu	neu	neu	pos
	MT	neu	pos	neu	pos	pos	neu
Memo from Frankfurt: Germany relives 1970s terror as 2 seek release from jail	GT	neu	neg	neg	neg	neg	neg
	ST	neg	neg	pos	neu	neg	neg
	MT	neu	neg	pos	neu	neg	neg
Luxury digs in South Carolina’s Lowcountry	GT	neu	neu	pos	neu	neu	neu
	ST	neg	pos	pos	neu	neu	neg
	MT	neu	pos	pos	neu	neu	neu

## Data Availability

COGNIMUSE Dataset, IEMOCAP Dataset, SemEval_2007 Dataset.
